# Supplementary material for: Curcumin Induces Homologous Recombination Deficiency by BRCA2 Degradation in Breast Cancer and Normal Cells
Source: Cancers (Basel). 2025 Jun 24;17(13):2109. doi: 10.3390/cancers17132109 (PMC12248657; doi:10.3390/cancers17132109)
Supplement: Supplementary file 1 [file cancers-17-02109-s001.zip › cancers-3707758-SI.pdf]

Supplementary

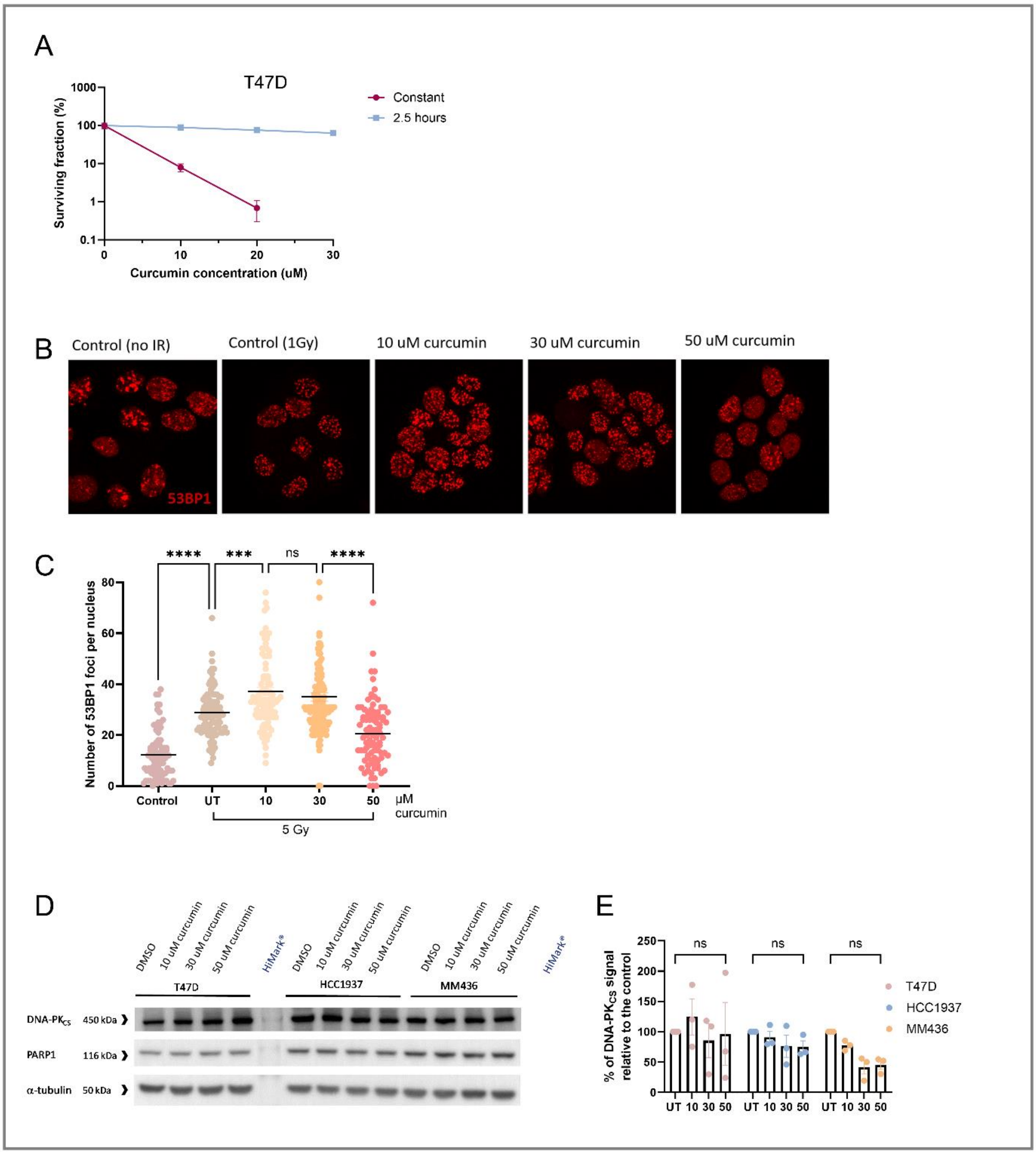

Supplementary Figure S1. The effects of curcumin on the survival, 53BP1 foci formation of the T47D cell line and DNA-PK$_{CS}$ levels in the T47D, HCC1937 and MM436 cell lines. (A) The results of the clonogenic survival assay of the T47D cell line after treatment with 10, 20 and 30 µM of curcumin for 2.5 hours of constantly. The surviving fraction is depicted in the graph with error bars indicating the SD. (B) Confocal images of the 53BP1 foci (red) with and/or without IR after treatment with increasing concentrations of curcumin. (C) Quantification of the images from figure B using an automated macro in the ImageJ software. Each data point

represents the number of 53BP1 foci per nuclei. Significance of the changes between the conditions is depicted with ns representing non-significant changes. (D) DNA-PK$_{CS}$ Western Blot results after treatment with curcumin of the T47D, HCC1937 and MM436 cell lines. (E) Quantification of the DNA-PK$_{CS}$ Western Blot, with each data point representing the relative band values for one replicate. Experiment was repeated in n=3.

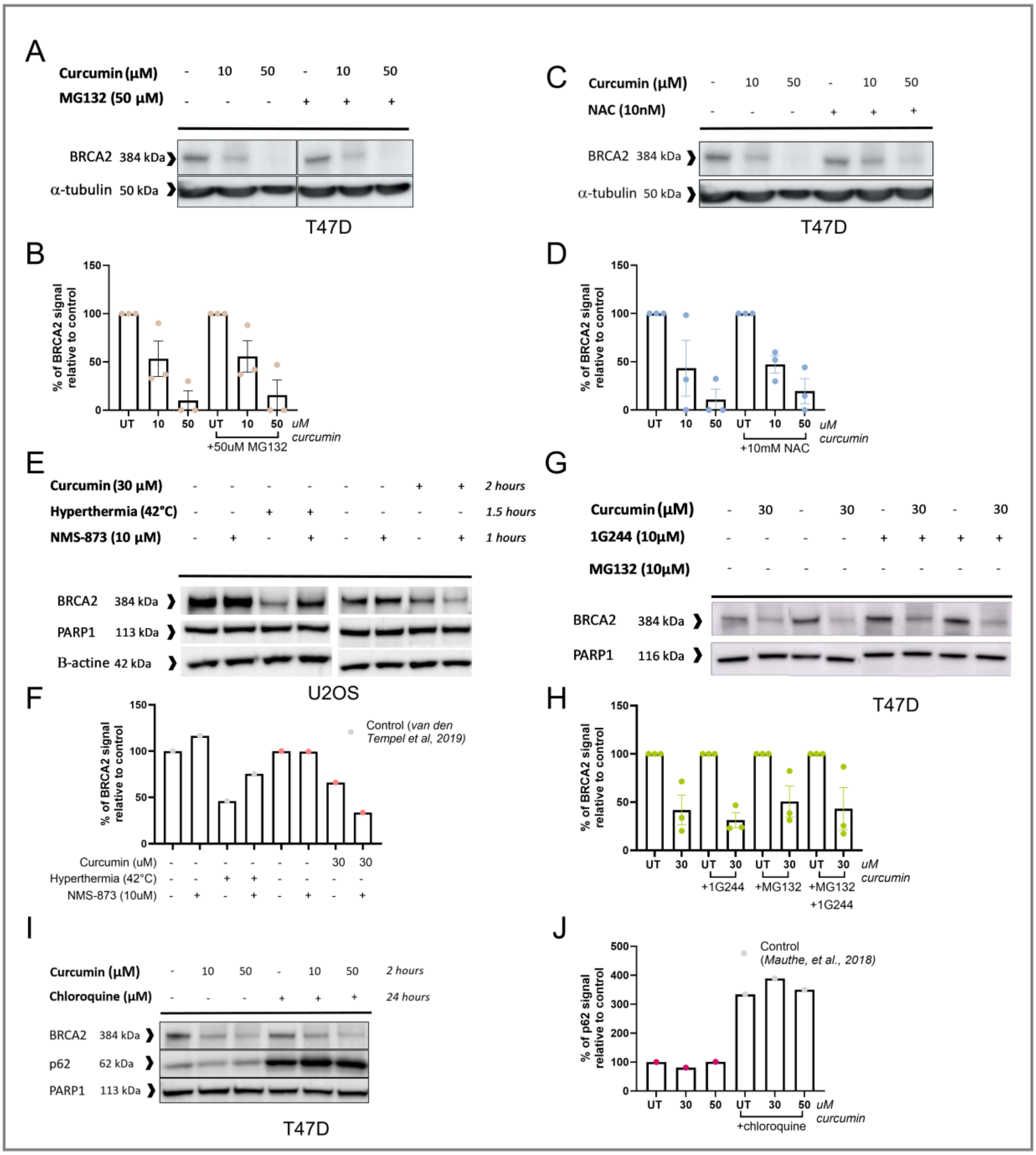

Supplementary Figure S2. Western Blot analysis investigating the mechanism behind BRCA2 degradation in the T47D cell line. (A, B) BRCA2 Western Blot and quantification data of the T47D cell line treated with curcumin alone and in combination with the proteasome inhibitor MG132. Each data point represents a relative band intensity per replicate with n=3. (C, D) BRCA2 Western Blot and quantification data of the T47D cell line treated with curcumin alone and in combination with the antioxidant NAC. Each data point represents a relative band intensity per replicate with n=3. (E, F) BRCA2 Western Blot and quantification data of the U2OS cell line treated curcumin alone and in combination with the p97 inhibitor NM-873. Internal

controls including hyperthermia treatment (42°C) were taken along with this experiment to ensure proper functioning of the inhibitor, as depicted in the figure. (G, H) BRCA2 Western Blot and quantification data of the T47D cell line treated with curcumin alone and in combination with the proteasome inhibitor MG132 and the DDP9 inhibitor 1G244. Each data point represents a relative band intensity per replicate with n=3. (I, J) BRCA2 and p62 Western Blot and quantification data of the T47D cell line treated with curcumin alone and in combination with the autophagy inducer chloroquine. This internal control was taken along with this experiment to ensure proper functioning of the inhibitor, as depicted in the figure.
